# Dependence of phase configurations, microstructures and magnetic properties of iron-nickel (Fe-Ni) alloy nanoribbons on deoxidization temperature in hydrogen

**DOI:** 10.1038/srep37701

**Published:** 2016-11-23

**Authors:** Panpan Jing, Mengting Liu, Yongping Pu, Yongfei Cui, Zhuo Wang, Jianbo Wang, Qingfang Liu

**Affiliations:** 1 School of Materials Science and Engineering, Shaanxi University of Science and Technology, Xi’an, 710021, People’s Republic of China; 2 Key Laboratory of Magnetism and Magnetic Materials of the Ministry of Education, Lanzhou University, Lanzhou, 730000, People’s Republic of China

## Abstract

Iron-nickel (Fe-Ni) alloy nanoribbons were reported for the first time by deoxidizing NiFe_2_O_4_ nanoribbons, which were synthesized through a handy route of electrospinning followed by air-annealing at 450 °C, in hydrogen (H_2_) at different temperatures. It was demonstrated that the phase configurations, microstructures and magnetic properties of the as-deoxidized samples closely depended upon the deoxidization temperature. The spinel NiFe_2_O_4_ ferrite of the precursor nanoribbons were firstly deoxidized into the body-centered cubic (bcc) Fe-Ni alloy and then transformed into the face-centered cubic (fcc) Fe-Ni alloy of the deoxidized samples with the temperature increasing. When the deoxidization temperature was in the range of 300 ~ 500 °C, although each sample possessed its respective morphology feature, all of them completely reserved the ribbon-like structures. When it was further increased to 600 °C, the nanoribbons were evolved completely into the fcc Fe-Ni alloy nanochains. Additionally, all samples exhibited typical ferromagnetism. The saturation magnetization (*M*_s_) firstly increased, then decreased, and finally increased with increasing the deoxidization temperature, while the coercivity (*H*_c_) decreased monotonously firstly and then basically stayed unchanged. The largest *M*_s_ (~145.7 emu·g^−1^) and the moderate *H*_c_ (~132 Oe) were obtained for the Fe-Ni alloy nanoribbons with a mixed configuration of bcc and fcc phases.

As an important noble-metal-free alloy system, Fe-Ni alloys with limited-dimensional structures have received much attention. It is not only owing to their outstanding soft magnetic properties including high saturation magnetization (*M*_s_), high permeability (*μ*), high Curie Temperature (*T*_c_) and low coercivity (*H*_c_) and energy loss^1^,^2^,^3^,^4^,^5^,^6^, but also owing to their widely applications in electromagnetic microwave absorption, magnetic refrigeration systems, magnetic recording devices, magnetic resonance imaging and sensors^7^,^8^,^9^. Generally, Fe-Ni alloys are basically classified into two categories based on the mass ratio of Fe: Ni. The Fe-Ni alloys with 50 ~ 80 at% Ni are known as permalloys, that with 30 ~ 40 at% Ni are knows as Invar alloys^10^,^11^. On the other hand, Fe-Ni alloys can also be distinguished as body-centered cubic (bcc) and face-centered cubic (fcc) alloys^12^. Hence it means that the above mentioned magnetic properties (*M*_s_, *μ, T*_c_ and *H*_c_) of Fe-Ni alloys are closely related to its composition and phase configuration. As reported in 2008, Vitta *et al*. showed that the *M*_s_ of nanocrystalline Fe_100−x_Ni_x_ decreased progressively with Ni content increasing^10^. In 1986, Chuang *et al*. pointed out that, for Fe-Ni system below 1200 K, the *T*_c_ of the bcc phase monotonously decreased with increasing Ni content and finally vanished at 60 at% Ni, while that of the fcc phase increased at first and then decreased by varying Ni content in the range of 15.1 ~ 100 at% and it reached about 879.5 K at 75 at% Ni^12^. Furthermore, it is noteworthy that the preparation condition and topographic structure (shape and size) are also significant to the magnetic nanomaterials. By controlling the oxidation kinetics, in 2013, Ucar *et al*. successfully tuned the *T*_c_ of the ball milled γ-Fe_70_Ni_30_ and γ-Fe_72_Ni_28_ nanoparticles (NPs), and severally calculated their refrigeration capacities to be about 470 and 250 J·kg at 5 T^6^. Moghimi *et al*. revealed in 2014 that the maximum magnetization of the Fe_63_Ni_37_ concave cubic (CC) NPs (85 emu·g^−1^) at 0.5 T was higher than that of the cuboctahedron (COh) NPs (40 emu·g^−1^) while the *H*_c_ for the CC NPs (100 Oe) was smaller than that for the COh NPs (188 Oe) although both of them had same composition^13^. The discrepancy was ascribed to different shapes and phase (fcc, bcc) combinations.

One-dimensional (1D) Fe-Ni alloys (nanowires, nanochains and nanofibers) recently are of particularly scientific and technical interest amongst the numerous magnetic nanostructures. This is surely attributed to their unique natures of effectively conquering the abnormally agglomeration between NPs and exhibiting many useful magnetic properties. For instance, in 2005, Liu *et al*. found that the *H*_c_ of the electrodeposited Fe_1-x_Ni_x_ nanowires assisted by AAO templates didn’t monotonically decrease as Ni content increased at 300 K^14^. In 2012, Jia *et al*.^15^ and Lu *et al*.^16^ respectively prepared FeNi_3_ nanochains through a template-free microwave-hydrothermal method and a CTAB mediated aqueous solution method to obtain the enhanced *H*_c_ and *M*_s_, respectively compared with that of bulk FeNi_3_ and monodispersed FeNi_3_ nanospheres. Lu also revealed that the imaginary permeability of the FeNi_3_ nanochains showed a broad resonance peak in the frequency range of 150 MHz to 4 GHz^16^. Thus it is believed that preparing a greater variety of 1D Fe-Ni alloy nanostructures with more satisfactory magnetic properties should be significantly meaningful.

To prepare 1D Fe-Ni alloy nanostructures, electrospinning technique is intensively recommended due to its simplicity, efficiency and versatility^17^,^18^,^19^. Earlier reports have certified that nanofibers^20^, nanotubes^21^ and nanoribbons^22^ could be directly obtained via the electrospinning route combined with a series subsequent treatments, and that all of these 1D nanostructures were tens of micrometers in length and their diameters or width can be flexibly tuned from nanometers to micrometers. To date, however, only Lee *et al*. prepared the Fe-Ni nanofibers using electrospinning route followed by air-calcination and H_2_-deoxidization, and studied the electromagnetic wave absorption property in the GHz frequency range^23^. Unlike nanofibers and nanotubes possessing round cross sections, on the other hand, novel nanoribbons are slightly different due to their rectangular cross sections. Nanoribbons could also be regarded as a development by cutting a finite-width slice from the two-dimensional nanosheets. In recently works, we have demonstrated that the electrospun SrFe_12_O_19_ and CoFe_2_O_4_ nanoribbons both exhibited deeply impressed magnetic properties^22^,^24^. To the best of our knowledge, there is still no public report on Fe-Ni alloy nanoribbons. Furthermore, the worth emphasizing is that this work is an indispensable part in research system of iron-based nanoribbons in the range of ferrite to iron-based alloys. Therefore our main objective in this study is to synthesize Fe-Ni alloy nanoribbons through the electrospinning route followed by air-annealing and H_2_-deoxidizing successively, and to systematically explore the effect of deoxidization temperature on their morphologies, phase configurations and room temperature magnetic properties.

## Experimental Section

### Synthesis of samples

Chemical agents of polyvinylpyrrolidone (PVP), nickel nitrate hexahydrate (Ni(NO_3_)_2_·6 H_2_O), iron nitrate nonahydrate (Fe(NO_3_)_3_·9 H_2_O) with analytical-grade were used without any purification. The Fe-Ni alloy nanoribbons were synthesized through an electrospinning route followed by air-annealing firstly and then H_2_-deoxidation successively. The detailed procedure is described as below. Firstly, 0.132 g Ni(NO_3_)_2_·6 H_2_O, 0.368 g Fe(NO_3_)_3_·9 H_2_O and 0.4 g PVP were sufficiently dissolved in a mixed solution of 1.5 g deionized water and 2.4 g C_2_H_5_OH under vigorous stirring for overnight to form a steady and viscous spinning solution. Secondly, the obtained solution was placed in a glass syringe equipped with a stainless steel needle (0.4 mm inner diameter). Subsequently, the electrospinning was carried out at a DC high-voltage of 15 kV, a spacing of 20 cm between the needle tip and metal collector, and a flow rate of 0.3 mL·h^−1^. Several hours later, the PVP/Fe(NO_3_)_3_/Ni(NO_3_)_2_ precursor nanoribbons were deposited on the collector with a form of an uniform membrane. Thirdly, the as-spun precursor membrane was aged initially at 60 °C in a drying box for a few hours followed by annealing at 450 °C for 2 h in air with a heating rate of 1 °C·min^−1^. Then the as-annealed products S0 was divided into four groups, which were further deoxidized at 300, 400, 500 and 600 °C in H_2_ atmosphere with a constant flow for 1 h respectively. Finally, the corresponding Fe-Ni alloy nanoribbons S1-S4 were obtained.

### Characterization of samples

The morphologies and microstructures of all samples were observed by field emission scanning electron microscopy (FESEM, Hitachi S4800, Japan) and transmission electron microscopy (TEM, FEI Tecnai^TM^ G^2^ F30, USA) equipped with an energy dispersive X-ray spectroscopy (EDX, Oxford Instrument, UK). The phase components, elements and corresponding chemical states were detected by powder X-ray diffraction on a X-ray diffractometer (XRD, Philips X’Pert Pro MPD, the Netherlands) with Cu-Kα irradiation (λ = 1.54056 Å), high-resolution transmission electron microscopy (HRTEM), EDX and X-ray photoelectron spectroscopy (XPS, Kratos AXIS Ultra^DLD^, 600 W, UK) with an monochrome Al-Kα probe beam. All the recorded XPS spectra were calibrated using C 1 s peak with the binding energy of 284.8 eV and were analyzed through Gaussian-fitting. A vibrating sample magnetometer (VSM, Lakeshore 7403, USA) was used to investigate the room temperature magnetic properties of the samples.

## Results and Discussion

### Phase configurations and microstructures of samples

**Figure 1:**
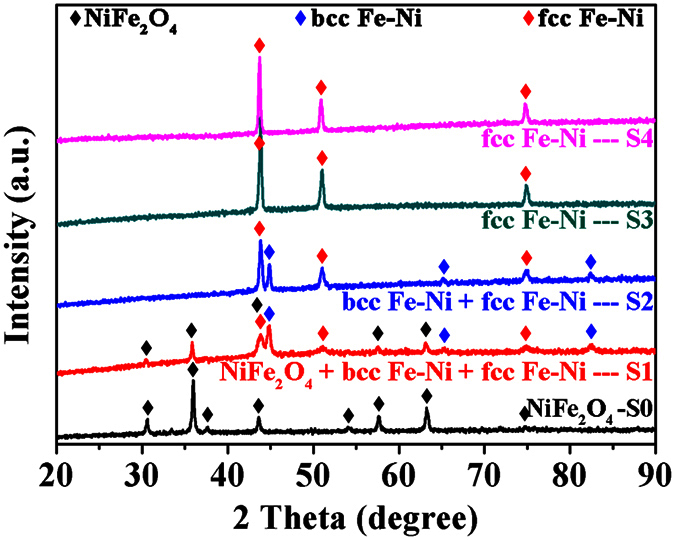
XRD patterns of the precursor sample S0 annealed at 450 °C in air and the as-deoxidized samples S1-S4 from S0 at 300, 400, 500 and 600 °C in H_2,_ respectively.

Figure 1 shows the XRD patterns of the H_2_-deoxidized samples S1-S4 as well as the air-annealed sample S0. For S0, the sample is certified as a single phase material. All the observed diffraction peaks marked by the black diamonds can be unambiguously indexed to the standard spinel NiFe_2_O_4_ with a cubic structure (JCPDS card no. 54-0964), and they are corresponded to the (200), (311), (400), (422), (511), (440), (622) and (533) planes, respectively. Neither amorphous materials nor other secondary phases are observed. When the pure NiFe_2_O_4_ (S0) is H_2_-deoxidized at 300 °C, it is clearly found that the diffraction peaks of NiFe_2_O_4_ are weakened but some new diffraction peaks emerged in the obtained sample S1. The peaks marked by the red diamonds at about 43.88°, 50.88° and 74.75° can be respectively indexed to (111), (200) and (220) planes of the fcc Fe-Ni alloy (JCPDS card no. 47-1405) and the peaks marked by the blue diamonds at about 44.76°, 65.16° and 82.4° can be respectively indexed to (110), (200) and (211) planes of the bcc Fe-Ni alloy (JCPDS card no. 37-0474). That is, NiFe_2_O_4_ in S0 is partially deoxidized to fcc and bcc Fe-Ni alloys in S1. Then sample S1 is a complex of the cubic NiFe_2_O_4_, fcc Fe-Ni and bcc Fe-Ni. However, the peak intensities of fcc Fe-Ni is weaker than that of bcc Fe-Ni. When S0 is H_2_-deoxidized at 400 °C, the peaks of NiFe_2_O_4_ phase disappear completely in sample S2, only the peaks of fcc and bcc Fe-Ni phases are observed and the peak intensities of fcc Fe-Ni are strengthened obviously. Thus sample S2 is a pure alloy just with a mixed phase of fcc and bcc Fe-Ni. When the deoxidization temperature is further risen to 500 °C, the peaks of fcc Fe-Ni strengthen further but that of bcc Fe-Ni disappear. Therefore, the as-deoxidized sample S3 is a pure fcc Fe-Ni alloy. Seeing from the phase transformation from S0 to S3, it can be concluded that the cubic NiFe_2_O_4_ is deoxidized to bcc Fe-Ni at first and then transformed into fcc Fe-Ni with the deoxidization temperature increasing. For S4 resulted from deoxidizing S0 at 600 °C, its phase is fcc Fe-Ni undoubtedly. Using Debye-Scherrer equation^25^, the average grain sizes (*D*) of fcc Fe-Ni alloy in S1, S2, S3 and S4 are estimated to be about 17.2 ± 0.30, 29.8 ± 0.32, 34.1 ± 0.27 and 39.9 ± 0.35 nm respectively. It indicates that the as-deoxidized fcc Fe-Ni alloy grains gradually grow with the increased deoxidization temperature.

**Figure 2:**
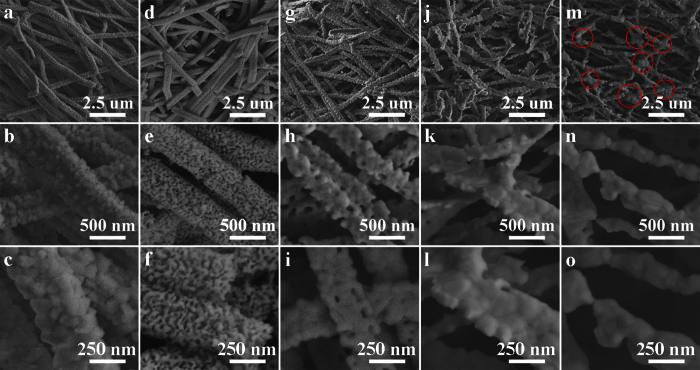
FESEM images of the precursor sample S0 (**a–c**) and as-deoxidized samples S1 (**d–f**), S2 (**g–i**), S3 (**j,l**) and S4 (**m–o**).

The morphology features of all samples S0-S4 with the defined phase configurations are examined by FESEM firstly. As presented in Fig. 2(a–c), a large number of continuously ribbon-like structures with random direction are found for sample S0 (NiFe_2_O_4_). The ribbon-width is ranged from 269 nm to 512 nm and the average width is about 348.4 nm. Each of nanoribbon is quite uniform in width along the length direction and is constructed by small nanoparticles (NPs). When the NiFe_2_O_4_ nanoribbons are deoxidized at 300 to 600 °C, some changes are also occurred on the microstructures beyond on the phase configurations of the resultant products, which are shown in Fig. 2(d–o). Firstly, in Fig. 2(d–f), when the deoxidization temperature is 300 °C, the obtained composite nanoribbons S1 of NiFe_2_O_4_ + fcc Fe-Ni + bcc Fe-Ni well keep the continuous structures of the NiFe_2_O_4_ nanoribbons. Secondly, when the temperature is risen up to 400 and 500 °C, the NPs contained within the composite nanoribbons S2 of fcc Fe-Ni + bcc Fe-Ni (Fig. 2(g–i)) and pure nanoribbons S3 of fcc Fe-Ni (Fig. 2(j–l)) increase sharply and fuse together. And the nanoribbons S3 become uneven and bent. Compared to S0, however, the average ribbon-widths of S1, S2 and S3 don’t change much and they are about 347.9, 345.1 and 345.4 nm respectively. Thirdly, when the deoxidization temperature is further increased to 600 °C, basically no nanoribbons except for NP-chain (or nanochains) are found in the final fcc Fe-Ni sample (S4) (Fig. 2(m–o)). Due to the excessive growth and fusion together of their NPs, the nanochains S4 are constructed by the NP-clusters as one-by-one and the average diameter is about 241 nm. Moreover, unlike the nanoribbons of S0-S3, the nanochains of S4 connect with each other by lots of junctions, which were marked by the red circles. Hence the whole sample S4 looks more like a three-dimensional network of the nanochains.

**Figure 3:**
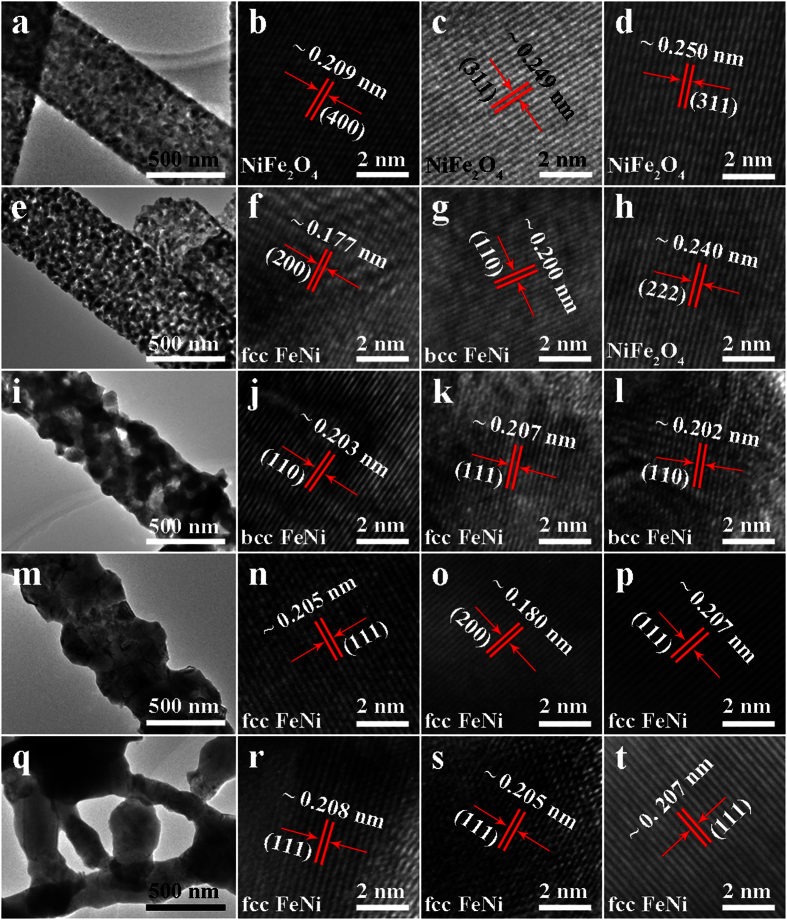
TEM and HRTEM images of the precursor sample S0 (**a–d**) and as-deoxidized samples S1 (**e–h**), S2 (**i–j**), S3 (**m–p**) and S4 (**q–t**).

TEM analysis was carried out to further explore the microstructures of S0-S4. Firstly, Fig. 3(a,e,i,m,q) describe the typical high-magnification TEM images of the random individuals of all samples. These closer TEM examinations powerfully certify the ribbon-like structures of S0-S3 and the chain-like structure of S4. Owing to the continued particle growth during the second annealing in H_2_, NPs of the deoxidized S1-S4 are bigger than that of their precursor nanoribbons S0. For S1-S4, the particle size increase gradually with the deoxidization temperature increasing. Secondly, Fig. 3(b–d,f–h,j–l,n–p,r–t) respectively display the corresponding HRTEM images. In Fig. 3(b–d), the lattice fringes scanned from the selected NPs of S0 are measured to be about 0.209, 0.249 and 0.250 nm, which are indexed well to (400), (311) and (311) planes of NiFe_2_O_4_ (JCPDS card no. 54-0964). In Fig. 3(f–h), the measured distances of about 0.177, 0.200 and 0.240 nm of S1 are approximately equivalent to the interlayer distances of (200), (110) and (222) planes of the fcc Fe-Ni alloy, bcc Fe-Ni alloy and spinel NiFe_2_O_4_, respectively. Thus it proves that the chemical phase of S0 nanoribbons is pure NiFe_2_O_4_ and the S1 nanoribbons are assembled by the fcc Fe-Ni, bcc Fe-Ni and spinel NiFe_2_O_4_ NPs. In other words, it further demonstrates that NiFe_2_O_4_ of S0 transforms to the complex of fcc Fe-Ni alloys, bcc Fe-Ni alloys and spinel NiFe_2_O_4_ ferrite after annealing in H_2_ at 300 °C for S1. Similarly, from Fig. 3(j–l,n–p,r–t), it also notifies that the nanoribbons S2 are constructed by the fcc and bcc Fe-Ni NPs, the nanoribbons S3 and nanochains S4 are both purely constructed by the fcc Fe-Ni NPs, respectively.

**Figure 4:**
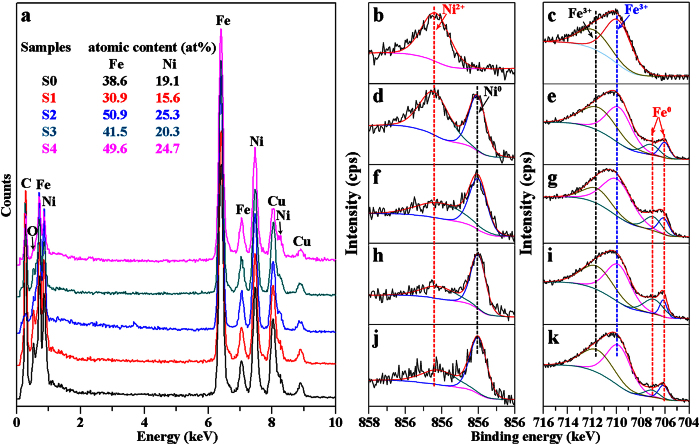
TEM-EDX patterns (**a**), high-resolution XPS spectra of Ni 2p_3/2_ and Fe 2p_3/2_ of the precursor sample S0 (**b,c**) and as-deoxidized samples S1 (**d,e**), S2 (**f,g**), S3 (**h,i**) and S4 (**j,k**).

To identify the chemical elements and corresponding surface chemical states of S0-S4, the TEM-EDX and XPS spectra are presented. With the exception of the extra signals of C and Cu elements descended from the carbon-coated copper grid clamping the samples for TEM measurements, as shown in Fig. 4a, the peaks of Fe, Ni and O elements are detected together in the spectra of all samples S0-S4. Of which, the atomic ratio of Fe: Ni (38.6: 19.1) of S0 is basically equivalent to the stoichiometric ratio (n(Fe): n(Ni) = 2: 1) of NiFe_2_O_4_. Although S1-S4 are respectively the complex of NiFe_2_O_4_ + fcc Fe-Ni + bcc Fe-Ni (S1), the complex of fcc Fe-Ni + bcc Fe-Ni (S2) and the pure fcc Fe-Ni (S3 and S4), their total atomic ratios of Fe: Ni (30.9: 15.6, 50.9: 25.3, 41.5: 20.3 and 49.6: 24.7) are still very close to 2: 1. Differently, however, the O contents are found to have a decrease in S2-S4 compared to S0 and S1. For S0 and S1, O elements are mainly belonged to the NiFe_2_O_4_, but the small amounts of O elements detected in S2-S4 are probably ascribed to the self-oxidized surfaces. This assumption will be carefully discussed as below. According to the obtained atomic ratios of Fe: Ni, additionally, the S2-S4 can be roughly expressed as Fe_67_Ni_33_. Fig. 4(b,d,f,h,j) and (c,e,g,i,k) reveal the recorded high-resolution XPS spectra of Ni 2p_3/2_ and Fe 2p_3/2_, respectively. All the binding energy scales are calibrated using the C 1 s peak with a binding energy of 284.8 eV. For precursor nanoribbons S0, the only peak located at about 854.50 eV in its Ni 2p_3/2_ spectrum (Fig. 4(b)) is assigned to the Ni^2+^ of O-Ni bonds^26^,^27^. The peak observed in Fe 2p_3/2_ spectrum (Fig. 4(c)) can be fitted into two peaks located at about 711.81 and 709.91 eV, which are respectively assigned to Fe^3+^ in tetrahedral-site and Fe^3+^ in octahedral-site of the inverse spinel NiFe_2_O_4_^28^,^29^. For reduced nanoribbons S1, besides the peak of Ni^2+^ with a higher binding energy presented in its Ni 2p_3/2_ spectrum (Fig. 4(d)), another peak with a lower binding energy at about 852.11 eV is also observed and assigned to the metallic Ni^26^,^27^,^30^. By fitting, similarly, its Fe 2p_3/2_ spectrum (Fig. 4(e)) contains four peaks, which can be fallen into two categories. The first and second peaks located at higher binding energies are surely assigned to Fe^3+^ of the NiFe_2_O_4_, but the third and fourth peaks located at lower binding energies of about 707.24 and 706.03 eV are both assigned to metallic Fe^29^,^31^,^32^. Based on the experiment process and above analysis, the metallic Ni and Fe should be derived from the partial deoxidized NiFe_2_O_4_ and they are further compounded to fcc and bcc Fe-Ni alloy NPs. For S2-S4 (Fe-Ni alloys), the peaks of Ni^2+^ and metallic Ni are also found in their Ni 2p_3/2_ spectra (Fig. 4(f,h,j)) as well as the peaks of Fe^3+^ and metallic Fe found in Fe 2p_3/2_ spectra (Fig. 4(g,i,k)). However, the peak signals of Ni^2+^ in S2-S4 are much weaker than that of metallic Ni while the peaks of Fe^3+^ are much stronger than that of metallic Fe. It means that the reduced Fe element is oxidized more easily than Ni element in air. Then Ni element in surfaces of S2-S4 mainly exists in metallic state but Fe element exists in oxide state. Because no any obvious Ni- or Fe-oxides are observed from the XRD patterns and HRTEM images of S2-S4, it suggests that the detected signals of Ni^2+^ and Fe^3+^ should be ascribed to the slightly oxidization shells on their surfaces.

### Magnetic properties of samples

**Figure 5:**
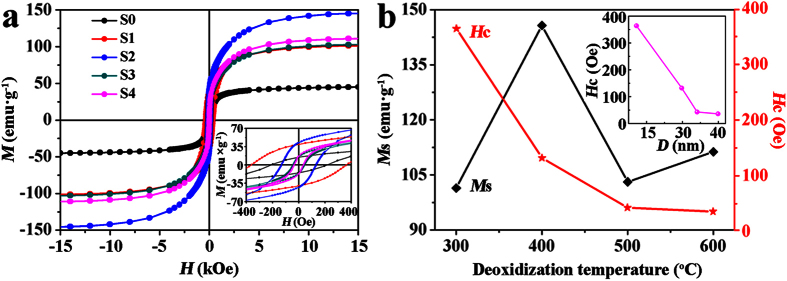
(**a**) *M-H* loops of S0-S4 recorded at room temperature. (**b**) *M*_s_ and *H*_c_ of S1-S4 as functions of the deoxidization temperature. The insets in (**a,b**) show the expended views of *M-H* loops and the *H*_c_ as a function of the grain size (*D*), respectively.

Figure 5(a) depicts the magnetization (*M*-*H*) loops of S0-S4 recorded at room temperature and the inset shows the corresponding enlarged views. It reveals that all samples exhibit typical ferromagnetic behaviors. For NiFe_2_O_4_ precursor nanoribbons (S0), the *M*_s_ and *H*_c_ are respectively about 45.1 emu·g^−1^ and 212 Oe. The hysteresis loops of the deoxidized S1-S4 closely vary with the deoxidization temperature. The largest *M*_s_ about 145.7 emu·g^−1^ is obtained for S2. For standard crystalline Fe and Ni, their magnetic moments per cell are about 2.2 μB and 0.6 μB^13^,^33^, respectively. And the bulk *M*_s_^34^ of bcc Fe is about 222 emu·g^−1^. Therefore, the bulk *M*_s_ of Fe_67_Ni_33_ can be approximately estimated to be about 167 emu·g^−1^, which is basically in constant with the reported value of 168 emu·g^−1^ in the literature^35^,^36^. In our case, thus, the experimental value is very close to the estimated bulk value. The above investigations of XRD and XPS have indicated that a thin oxide shells present at the surface, in which the magnetic spins are disordered and pinned^37^,^38^. This could account for the slight difference of *M*_s_ between our result and the calculated bulk value. On the other hand, except for S1, the *H*_c_ values of S2-S4 (pure Fe_67_Ni_33_) are all lower than 150 Oe. Based on the above results, hence, the Fe-Ni alloy nanoribbon is a considerable soft magnetic nanomaterial. Additionally, the most significant feature of the Fe-Ni alloy nanoribbons is their unique geometry of the ribbon-like structure self-assembled by NPs. Compared with the zero-dimensional alloy materials, Fe-Ni alloy nanoribbons have an amazing advantage is that they can be used in the miniaturizing and high density electronic components such as antennas and stealth devices. Therefore, the high-frequency properties can probably be a major future research content and direction.

To further understand the effect of deoxidization temperature on the magnetic properties of S1-S4, the *M*_s_ and *H*_c_ values as functions of the deoxidization temperature are shown in Fig. 5(b). It is found that *M*_s_ of these Fe-Ni alloy samples firstly increases, then decreases, and finally increases with the deoxidization temperature increasing, but the *H*_c_ monotonously decreases firstly and then basically stays unchanged. To understand this, the plausible contributing reasons are clarified. For *M*_s_, firstly, because the deoxidization temperature of 300 °C in H_2_ for S1 is too low to completely deoxidize NiFe_2_O_4_ of S0 into Fe-Ni alloys but that of 400 °C for S2 is big enough to deoxidize NiFe_2_O_4_ into Fe-Ni alloys completely, a certain amount of residual NiFe_2_O_4_ in S1 certainly pulls down the total *M*_s_. Besides, the grain size of S1 (*D*_1_ ≈ 17.2 nm) is much smaller than that of S2 (*D*_2_ ≈ 29.8 nm). Hence even if S2 has a mix combination of fcc and bcc Fe-Ni phases, its *M*_s_ is still increased considerably compared to that of S1. Secondly, when the deoxidization temperature is risen up to 500 °C for S3, bcc Fe-Ni phase is transformed into fcc Fe-Ni phase and then the obtained S3 is pure fcc Fe-Ni with an increased grain size (*D*_3_) of about 34.1 nm. But, the increased grain size does not lead the decrease of *M*_s_. Then the decreased *M*_s_ of S3 mainly caused by vanishing of bcc Fe-Ni phase. This phenomenon is similar with Moghimi’s results about Fe_63_Ni_37_^13^. Moghimi and other works have pointed out that the *M*_s_ of bcc phase is universally larger than that of fcc phase in Invar Fe-Ni alloys (30 ~ 38 at% Ni)^13^,^39^,^40^, and that if the Fe-Ni alloy is a combination of bcc and fcc phases, its *M*_s_ linearly decreases with the fcc phase increasing^13^. Then S3 has a lower *M*_s_ than that of S2. Thirdly, when the temperature is sequentially increased to 600 °C, the composition of obtained S4 is still fcc Fe-Ni. Due to the same composition of Fe_67_Ni_33_ and same phase configuration, the different grain size of S4 (*D*_4_ ≈ 39.9 nm) and S3 (*D*_3_ ≈34.1 nm) is the only striking difference, which finally gives rise to the slight increase of *M*_s_. As same as *M*_s_, on the other hand, the variation of *H*_c_ is also related to the composition and grain size. Firstly, S1 is the composite of NiFe_2_O_4_ + fcc Fe-Ni + bcc Fe-Ni. Since the magneto-crystalline anisotropy constant (*K*_1_) of cubic NiFe_2_O_4_ (~10^5^ J·m^3^) is larger than that of either fcc or bcc Fe-Ni alloys (~10^3^ J·m^−3^)^41^,^42^,^43^, the exchange interactions between the NiFe_2_O_4_ and Fe-Ni NPs contribute to the largest *H*_c_ value (364 Oe) of S1 among the four samples. Secondly, both of our prepared Fe-Ni alloy nanoribbons (S2 and S3) and nanochains (S4) are self-assembled by Fe-Ni NPs. For simplicity sake, we can temporarily ignore the anisotropic difference originated from the slightly different shapes of nanoribbons and nanochains donating to *H*_c_. Thus we utilize the *H*_c_ of Fe-Ni NPs to approximately illustrate the *H*_c_ variation from S2 to S4. The random anisotropy model (RAM) originally proposed by Alben *et al*. reveals that the *H*_c_ of ferromegnetically coupled nanocrystallines is related to the average grain size (*D*) and ferromagnetic exchange length *L*_ex_ (*L*_ex_ = (*A*/*K*_1_)^1/2^, *A* and *K*_1_ respectively denote the exchange stiffness and the magneto-crystalline anisotropy constant^44^,^45^. If *D* < *L*_ex_, the *H*_c_ is compatible with *D*^*6*^ dependence. If *D* > *L*_ex_, the *H*_c_ is proportional to 1/*D*. To be emphasized, the RAM law for *H*_c_ is well-known for polycrystalline Fe-based alloys^46^,^47^,^48^. In Wen’s work, through a liner fitting of *A* and *K*_1_ values of bcc Fe and fcc Ni, he calculated not only the *A* and *K*_1_ values of Fe_70_Ni_30_ alloys but also the *L*_ex_ value of Fe_70_Ni_30_ (~ 14 nm)^45^. Using the suggested method, similarly, we also respectively estimate the *A, K*_1_ and *L*_ex_ of Fe_67_Ni_33_ alloys to be about 6.68 × 10^–12^ J·m^−1^, 3.18 × 10^4^ J·m^−3^ and 14.5 nm. In our case, hence, all of the average grain sizes of Fe_67_Ni_33_ NPs of S2-S4 are larger than the *L*_ex_. Furthermore, the inset in Fig. 5(b) shows that the variation of *H*_c_ with the grain size (*D*) from S1 to S3 except for S4 generally conforms to the *H*_c_ ∝ 1/*D* situation of the RAM theory. Therefore, the decrease of *H*_c_ from S1 to S2 to S3 is caused by the increase of grain size. Although all deoxidized samples are self-assembled by the Fe-Ni NPs, thirdly, the nanochains of S4 are still slightly different from the nanoribbons of S2-S3. On one hand, the nanochains are connected by the big clusters of Fe-Ni NPs. Within these clusters range, Fe-Ni NPs contact with each other tightly. That way, the exchange interactions between Fe-Ni NPs are increased at a certain extent. On the other hand, most of the nanochains of S4 connect with each other through the junctions, being marked by the red circles in Fig. 2m, but the nanoribbons of S2 and S4 are more isolated individuals. So there are still increased exchange interactions between the nanochains in S4 compared to S2 and S3. Hence the above two aspects of increased exchange interactions in S4 effectively compensate the decrease of *H*_c_ caused by the increase of grain size, and then the net *H*_c_ dose not decrease. Consequently, it is concluded that the deoxidization temperature has a great influence on the microstructures of Fe-Ni samples and then further affects the magnetic properties.

## Conclusions

Briefly, Fe-Ni alloy nanoribbons were successfully synthesized through an electrospinning route followed by air-annealing and H_2_-deoxidizing orderly. The results revealed that the deoxidization temperature had a significant influence on the phase components, morphologies and magnetic properties of the final samples. On the one hand, the air-annealed cubic NiFe_2_O_4_ ferrite could be firstly deoxidized into bcc Fe-Ni alloy and then transformed into fcc Fe-Ni alloy in H_2_ with the deoxidization temperature increasing. When the temperature is in the range of 300~500 °C, all the obtained samples possessed the ribbon-like structures perfectly. While when the temperature was further risen to 600 °C, the Fe-Ni alloys nanoribbons were evolved into the Fe-Ni alloy nanochains. On the other hand, the Fe-Ni alloys nanoribbons and nanochains both performed excellent ferromagnetism. Their *M*_s_ first increased, then decreased, and finally increased with the deoxidization temperature increasing, but their *H*_c_ monotonously decreased firstly and then basically stayed unchanged. Last but not least is that these results can provide a meaningful reference to related fundamental research and practical application in future.

## Additional Information

**How to cite this article**: Jing, P. *et al*. Dependence of phase configurations, microstructures and magnetic properties of iron-nickel (Fe-Ni) alloy nanoribbons on deoxidization temperature in hydrogen. *Sci. Rep.* **6**, 37701; doi: 10.1038/srep37701 (2016).

**Publisher's note:** Springer Nature remains neutral with regard to jurisdictional claims in published maps and institutional affiliations.
